# Utility of CD4 cell counts for early prediction of virological failure during antiretroviral therapy in a resource-limited setting

**DOI:** 10.1186/1471-2334-8-89

**Published:** 2008-07-04

**Authors:** Motasim Badri, Stephen D Lawn, Robin Wood

**Affiliations:** 1 The Desmond Tutu HIV Centre, Institute for Infectious Disease and Molecular Medicine, Faculty of Health Sciences, University of Cape Town, Cape Town, South Africa; 2 Clinical Research Unit, Department of Infectious and Tropical Diseases, London School of Hygiene & Tropical Medicine, London, UK

## Abstract

**Background:**

Viral load monitoring is not available for the vast majority of patients receiving antiretroviral therapy in resource-limited settings. However, the practical utility of CD4 cell count measurements as an alternative monitoring strategy has not been rigorously assessed.

**Methods:**

In this study, we used a novel modelling approach that accounted for all CD4 cell count and VL values measured during follow-up from the first date that VL suppression was achieved. We determined the associations between CD4 counts (absolute values and changes during ART), VL measurements and risk of virological failure (VL > 1,000 copies/ml) following initial VL suppression in 330 patients in South Africa. CD4 count changes were modelled both as the difference from baseline (ΔCD4 count) and the difference between consecutive values (CD4 count slope) using all 3-monthly CD4 count measurements during follow-up.

**Results:**

During 7093.2 patient-months of observation 3756 paired CD4 count and VL measurements were made. In patients who developed virological failure (n = 179), VL correlated significantly with absolute CD4 counts (r = - 0.08, *P *= 0.003), ΔCD4 counts (r = - 0.11, *P *< 0.01), and most strongly with CD4 count slopes (r = - 0.30, *P *< 0.001). However, the distributions of the absolute CD4 counts, ΔCD4 counts and CD4 count slopes at the time of virological failure did not differ significantly from the corresponding distributions in those without virological failure (*P *= 0.99, *P *= 0.92 and *P *= 0.75, respectively). Moreover, in a receiver operating characteristic (ROC) curve, the association between a negative CD4 count slope and virological failure was poor (area under the curve = 0.59; sensitivity = 53.0%; specificity = 63.6%; positive predictive value = 10.9%).

**Conclusion:**

CD4 count changes correlated significantly with VL at group level but had very limited utility in identifying virological failure in individual patients. CD4 count is an inadequate alternative to VL measurement for early detection of virological failure.

## Background

Access to antiretroviral therapy (ART) is expanding in low- and middle-income countries with over 2 million people receiving treatment by December 2006, representing 28% of the 7.1 million estimated to be in need [1]. Recent studies from sub-Saharan Africa have shown that ART is a cost-effective public health intervention [2-4]. Over 1.3 million people in the region were receiving ART by December 2006 and yet more than 3.5 million further individuals remained untreated [1]. To date, early pessimism that ART could not be effectively delivered on a large scale in the region using a simplified public health approach has proven largely unfounded. However, lack of laboratory monitoring to identify patients failing treatment and requiring a switch in treatment regimen remains a critical issue.

Plasma viral load (VL) monitoring, the gold standard used in high-income countries for diagnosing virological failure, is not available in many resource-limited settings. Currently a single World Health Organisation (WHO)-recommended second-line regimen is the only therapeutic option available for HIV-infected patients in sub-Saharan Africa who develop virological failure during their first-line regimen [5]. Although these regimens are offered free of charge in the national ART programme in some countries, no further treatment options are typically available in the public sector thereafter. Sensitive and specific means for timely identification of treatment failure are therefore greatly needed to maximize the benefits of these limited drug options.

Routine VL monitoring in resource-limited settings requires significant infrastructure and expertise and remains prohibitively expensive in most settings. Other low-cost means of detecting virological failure must therefore be considered. Colebunders and colleagues, for example, proposed an algorithm based on clinical and treatment history and inexpensive laboratory indices such as haemoglobin level and total lymphocyte count [6]. However, when evaluated in a South African cohort, the sensitivity and specificity of the algorithm were unacceptably low [7]. WHO has recommended use of CD4 cell count measurements and clinical outcomes for monitoring ART in the absence of VL [5]. However, the clinical and CD4 cell count changes that are able to predict virological failure have not been identified.

When considering the utility of CD4 cell counts as a surrogate for virological failure, the critical issue is whether the variability in CD4 cell count measurements adequately reflects the variability in viral load. A number of previous observations suggest that this may be limited. Firstly, in a study of untreated patients in the USA, higher VLs were associated with greater rates of CD4 cell decline at a group level, but had minimal value for predicting the rate of CD4 cell decline in individual patients; only 4%–6% of the variability in CD4 cell losses could be explained by plasma VL [8]. Secondly, it is well recognised that a significant proportion of patients receiving ART have discrepant virological and immunological responses. Blood CD4 cell counts fail to increase in 5%–50% of patients receiving ART despite prolonged undetectable plasma VL. Conversely, marked increases in CD4 cell counts are observed in some patients despite incomplete virological suppression [9-14]. Thirdly, in a study from Botswana, initial blood CD4 cell count increases only had moderate discriminative ability for identifying those patients who successfully achieved VL suppression after starting ART [15]. Collectively these existing data suggest that CD4 cell counts have limited capacity to explain the variability of VL measurements at an individual level both in treated and untreated patients.

A number of studies have previously examined factors associated with virological treatment failure in high-income settings [16-23]. However, the practical utility of CD4 cell count measurements as a substitute for viral load monitoring has not been specifically assessed using rigorous analyses. Data relevant to ART programmes in resource-limited settings are especially needed. We therefore conducted an analysis of longitudinal data from the Cape Town AIDS Cohort (CTAC) in South Africa in which CD4 cell counts and VL measurements are routinely measured every three months. Using all data points measured during follow-up, we determined the association between VL measurements, risk of virological failure and CD4 cell counts analysed as either absolute values, changes from baseline (ΔCD4 count) or the difference between consecutive values (CD4 cell count slope). We were thereby able to assess the utility of CD4 cell counts to predict virological failure in a resource-limited setting.

## Methods

### Setting and study population

The Cape Town AIDS Cohort (CTAC) has been described in detail previously [24]. In brief, ART-naïve patients were referred to the cohort from a wide range of primary health care facilities in Cape Town to the adult HIV clinics affiliated with the University of Cape Town (UCT). Patients accessed ART through participation in multicentre phase III clinical trials at the New Somerset Hospital and the Desmond Tutu HIV Research Centre at UCT between 1996 and 2006. Participants gave informed consent and clinical trials protocols were approved by the UCT Clinical Research Ethics Committee. Enrolment criteria differed between the various trials but collectively encompassed patients with a wide spectrum of baseline blood CD4 cell counts, viral load and clinical stages. All patients received a minimum of three antiretroviral drugs: a non-nucleoside reverse transcriptase inhibitor and two nucleoside analogues; three nucleoside analogues; or a protease inhibitor with two nucleoside analogues.

Viral load was determined by reverse transcriptase-polymerase chain reaction (Amplicor^®^, Roche Molecular Systems, Branchburg, New Jersey, USA) and CD4 counts were measured by flow cytometry (Beckman Coulter^®^, Miami, Florida, USA). Blood CD4 cell counts and plasma VL were measured every 2–3 months when patients were routinely reviewed. Clinical stage of disease was assessed using WHO criteria. Demographic data were recorded and the socioeconomic status of each patient was defined using the Cape Metropolitan Council suburbs composite index, which has been described previously [24].

### Statistical analyses

In all analyses conducted in this study, virological suppression was defined by a VL of < 400 HIV RNA copies/ml following initiation of ART. The baseline CD4 cell count was that measured at the time that virological suppression was first achieved. Virological failure was defined as the first episode of viral load ≥ 1,000 HIV RNA copies/ml following previous successful VL suppression, confirmed by a second consecutive measurement. To investigate sensitivity thresholds, we also explored in separate analyses VL thresholds of > 400 and of > 10,000 HIV RNA copies/ml. Changes in CD4 cell count were reported in two ways: ΔCD4 was defined as the change in CD4 cell count from the baseline value and the CD4 count slope was defined as the difference between consecutive CD4 cell count measurements as determined by subtraction of the former value from the latter value.

### Determinants of virological failure

The Wilcoxon matched pairs test was used to compare continuous variables and the χ^2 ^test for comparison of categorical variables. The Kaplan-Meier method was used to estimate the virological failure-free proportion. Cox proportional hazard regression models were fitted to identify factors associated with the likelihood of virological failure, using the SAS phreg procedure (SAS software version 8.2, SAS, Cary, NC, USA). In this analysis virological failure-free survival was defined as the time from the date of first virological suppression to when viral load was confirmed to reach > 1,000 copies/ml, death or last known clinic visit. Risk factors considered in the analysis were prevalent AIDS (prior to, or at the date of a first viral load < 400 HIV RNA copies/ml) and incident AIDS (occurring subsequent to the date of a first viral load < 400 HIV RNA copies/ml), socio-demographic variables (including age, socioeconomic status and gender), baseline CD4 cell count and follow-up CD4 cell count (categorized a priori as a < 100 or ≥ 100 cells/μl increase at any time-point during follow-up). Follow-up CD4 cell count measurements were modelled as a time varying covariate. At each time-point in the modelling process, the CD4 cell count value considered was the value recorded at that specific time-point, if available. Otherwise, the most recent recorded value (within 2–3 months) was considered. Variables significantly associated with the likelihood of occurrence of virological failure in univariate models (*P *< 0.05) were considered for inclusion in a multivariate model.

### Association between CD4 count and viral load failure

Different strategies were employed to comprehensively assess the strength of the association between treatment-induced changes in CD4 cell count and virological failure. Firstly, for patients who failed virologically, we fitted three separate scatter-plots of all VL measurements (log_10 _copies/ml) done during follow-up and either the concurrently measured absolute CD4 counts, ΔCD4 count values or CD4 count slopes at each time-point. In these analyses the strength of association was assessed by calculating Pearson correlation coefficients.

For patients who developed virological failure, we next compared the distributions of CD4 cell values, ΔCD4 counts and CD4 cell slopes measured at the time of failure with the distributions of all data points from patients who did not develop virological failure. All CD4 cell count and viral load values included in these analyses were concurrently measured during follow-up. Data were included from the date of first viral load suppression until the date of development of virological failure or the date of last CD4 count measurement for those who did not fail virologically.

We next determined the association between the CD4 cell count slope and virological failure using a receiver operating characteristic (ROC) curve. The area under the ROC curve was assessed with the use of the C statistic. Sensitivity, specificity, positive predictive value, negative predictive value estimates were calculated, with 95% confidence interval (CI), using Clopper-Pearson exact method or Fleiss approximation as appropriate.

## Results

### Virological failure during follow-up

Of 360 patients who started ART during the study period, 330 (91.7%) achieved initial viral load suppression during follow-up and were therefore included in the analyses of virological failure. All treatment regimens incorporated at least 3 drugs; the numbers of patients receiving regimens based on triple nucleosides, a non-nucleoside reverse transcriptase inhibitor or a protease inhibitor were 51 (15%), 115 (35%) and 164 (50%), respectively. Patients were followed for a median of 24.7 patient-months (IQR, 4.7–51.6) of observation. During this time, 15 (4.5%) patients died.

**Figure 1:**
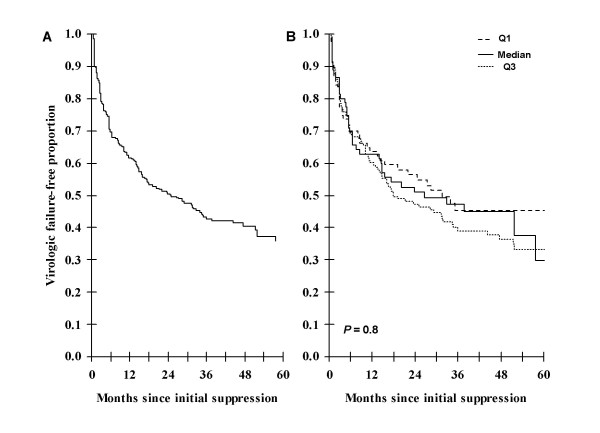
**(A) Kaplan-Meier probabilities of virologic failure-free proportion.** The numbers of patients followed up for 0, 12, 24, 36, 48 and 60 months were 330, 180, 123, 82, 39 and 26, respectively. (B) Kaplan-Meier probabilities of failure-free survival stratified by baseline CD4 cell count quartile range (median = 327; IQR = 205–435 cells/ul).

Overall, a total of 3756 paired CD4 cell count and VL measurements were made during 7093.2 patient-months of observation. 179 (54.2%) patients developed virological failure with an incidence of 30.3 (95%CI 26.2–34.2) cases per 100 patient-years. Virological suppression was maintained in the remaining 151 (45.8%) patients. Kaplan-Meier analysis showed that risk of virological failure decreased with increasing duration of follow-up (Figure 1A). The median time to development of failure was 24.7 months.

### Determinants of virological failure

The baseline clinical and socio-demographic characteristics are reported in Table 1. Groups of patients who did or did not develop virological failure were both composed of young adults with similar distributions of gender, socioeconomic status, and baseline CD4 cell count and clinical stage of disease. All had sexually acquired disease.

**Table 1:** Baseline demographic and clinical characteristics of the cohort studied (N = 330 patients).

Characteristic	Not failed (n = 151)	Failed (n = 179)	*P*-value
Gender			
Male	84(56)	93(52)	0.51
Female	67(44)	86(48)	
Age [median years(IQR)]	34(29–40)	32(27–38)	0.15
Socioeconomic status			
High status	68(45)	88(49)	0.45
Low status	83(55)	91(51)	
Baseline CD4 cell count (cells/μl)			
Median (IQR)	331(193–467)	323(208–431)	0.74
< 200	40(27)	38(21)	0.28
200–350	41(27)	62(35)	
> 350	70(46)	79(44)	
WHO stage			
Stage 1&2	74(49)	83(46)	0.82
Stage 3	59(39)	71(40)	
Stage 4	18(12)	25(14)	

In univariate Cox proportional hazards regression models, none of the variables examined was significantly associated with the likelihood of virological failure (Table 1). These variables included follow-up CD4 cell count (Wald test *P *= 0.32), baseline CD4 cell count (Wald test *P *= 0.46), baseline WHO stage (Wald test *P *= 0.50), incident AIDS (Wald test *P *= 0.22), age (Wald test *P *= 0.09), gender (Wald test *P *= 0.63), and socio-economic status (Wald test *P *= 0.53). The lack of association with baseline CD4 cell count was further confirmed using a stratified Kaplan-Meier plot (Figure 1B).

In view of the lack of significant associations between patient characteristics and virological failure, multivariate analysis was not done. Collectively these data showed that development of virological failure was not associated with baseline patient characteristics, follow-up CD4 cell counts or the development of new AIDS-defining illnesses. In separate analyses, use of VL thresholds of > 400 and of > 10,000 HIV RNA copies/ml produced the same outcomes.

### CD4 cell count changes and virological failure

**Figure 2:**
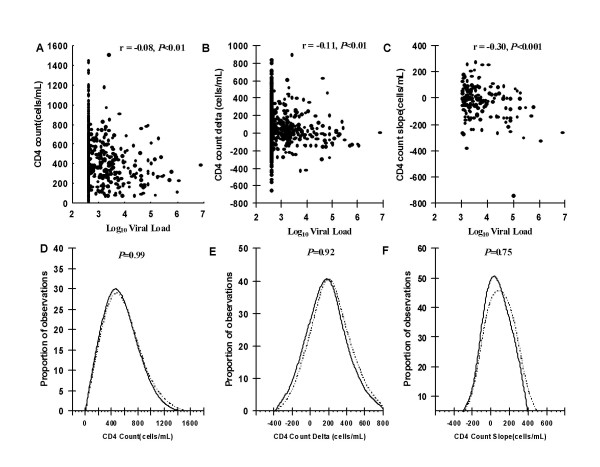
**Scatter plots of (A) absolute CD4 cell count, (B) ΔCD4 cell count (change in CD4 count from baseline) and (C) CD4 cell count slope (difference between consecutive CD4 count measurements) and corresponding viral load values (log_10 _copies/ml) measured in patients who developed virological failure.** Distributions of (D) absolute CD4 counts, (E) ΔCD4 counts and (F) CD4 count slopes of patients (n = 179) at the time of virological failure (dashed lines) compared to the distribution of measurements of all patients (n = 330) at all time-points when viral load remained suppressed (solid lines).

We next examined in greater detail the associations between all viral load and CD4 cell count values measured concurrently during follow-up. Correlations between VL and absolute CD4 count, ΔCD4 cell counts and CD4 cell slopes were calculated for those patients who developed virological failure (Figure 2A–C). Significant correlations were observed between log_10 _VL and both absolute CD4 cell count values (r = - 0.08, *P *< 0.01), ΔCD4 cell count (r = - 0.11, *P *< 0.01), and most strongly with CD4 count slope (r = - 0.30, *P *< 0.001). This suggests that the CD4 count slope at a given time-point would be the strongest indicator of the likelihood of virological failure.

CD4 cell counts measured at the time of virological failure were also compared with the distribution of all CD4 cell count measurements obtained from patients who did not develop failure (Figure 2D–F). These analyses showed that the distributions of absolute CD4 counts, ΔCD4 counts and CD4 count slopes did not significantly differ comparing values during virological failure to values during viral load suppression (*P *= 0.99, *P *= 0.92 and *P *= 0.75, respectively).

**Figure 3:**
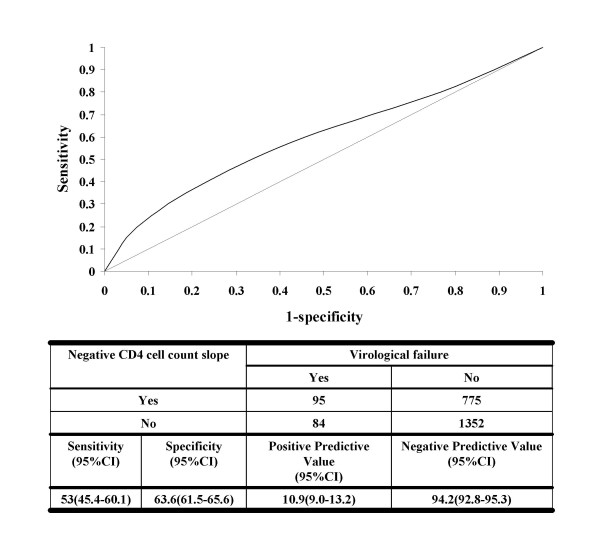
Receiver Operating Characteristic (ROC) curve assessing the association between a negative CD4 cell count slope (ie a falling CD4 count) and virological failure (area under the curve = 0.59).

Since CD4 slopes were the parameter most strongly associated with log_10 _VL among those who developed virological failure, we fitted a receiver operating characteristic curve (ROC) using data from all the patients to examine this association further (Figure 3). This analysis showed that the predictive value of CD4 cell slope for virological failure was poor. The area under the ROC curve was 0.59 and the sensitivity, specificity, positive predictive and negative predictive values were all low (Figure 3).

### CD4 cell counts among patients who did not achieve virological suppression

**Figure 4:**
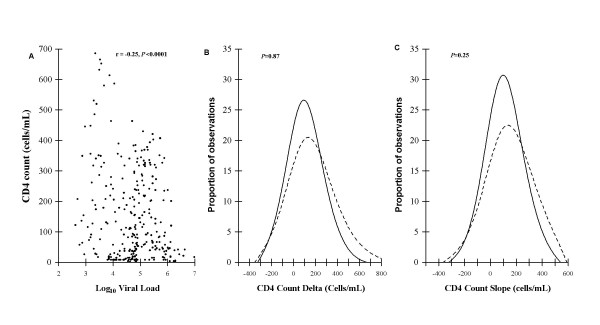
**Scatter plot of the absolute CD4 count (cells/μl) and corresponding HIV RNA viral load (log_10 _copies/ml) for the subset of patients (n = 30) who did not ever achieve virological suppression during ART (A).** The distributions of ΔCD4 counts (B) and CD4 cell count slopes (C) of this group are compared with that of patients who achieved and maintained virological suppression throughout the study period (n = 151).

Virological suppression was not achieved by 30 (8.3%) of the total of 360 patients treated in this cohort during follow-up and were therefore not included in the above analyses. Separate analysis of data from these patients showed that a significant correlation was similarly observed between all measurements of absolute CD4 counts and the corresponding log_10 _VL measurements (r = - 0.25, *P *< 0.0001) (Fig 4A). However, the distributions of the ΔCD4 counts and CD4 count slopes in this group did not differ significantly from those observed among the 151 patients who achieved and maintained virological suppression during follow-up (*P *= 0.87 and *P *= 0.25 respectively) (Fig 4B–C). This showed that CD4 cell counts were also a poor correlate of viral load among patients who did not achieve viral load suppression.

## Discussion

Early detection of virological failure is important for optimal management of HIV-infected patients receiving ART. Patients who continue to receive a failing regimen are at risk of immunological failure, morbidity and death. Moreover, accumulation of multiple antiretroviral drug resistance mutations may compromise the response to future drugs and fuel the spread of primary drug resistance within communities. Since VL monitoring is not available in most resource-limited settings, we investigated the utility of CD4 cell count measurements for predicting virological failure in a cohort of South African patients. Baseline absolute CD4 cell counts as well as clinical and socio-demographic characteristics were not predictive of virological failure. Analyses of longitudinal data from those who developed virological failure revealed that absolute CD4 cell counts and CD4 cell count changes (ΔCD4 cell counts and CD4 cell count slopes) were significantly correlated with viral load measurements at a group level. However, subsequent analysis showed that none of these methods of analysing CD4 cell counts could be used to identify individual patients at the time they developed virological failure. Since the distributions of CD4 cell counts and CD4 cell count changes among those with virological failure did not differ significantly from those of patients who maintained virological suppression, these could not be used to provide a clinically useful means for individual patient assessment for virological failure.

A unique feature of our study is that we used a novel modelling approach that accounted for all CD4 cell count and VL values measured during follow-up from the first date that VL suppression was achieved. Some previous studies have modelled the difference between the CD4 cell counts measured at initiation of treatment and at a single arbitrary point during ART defined *a priori*. Other studies assessing factors associated with virological failure did not account for all CD4 cell count measurements performed during follow-up [15-23]. Neither of these approaches fully evaluates CD4 cell count dynamics during ART, increasing the potential for misclassification bias. Our analytic approach also differs in that we modelled virological failure as the end-point of interest rather than virological treatment success as reported elsewhere. We used a VL > 1,000 copies/ml to define treatment failure consistent with local protocols. However, the same outcomes were obtained using thresholds of > 400 and > 10,000 copies/ml, which is consistent with previous data from this setting [25].

Baseline CD4 cell count was not predictive of virological failure in this ART-naïve population. However, in patients who developed virological failure, absolute CD4 cell count measurements, ΔCD4 cell counts and CD4 cell count slopes during ART each correlated significantly with VL measurements taken at the same time-points. Of these three parameters, the CD4 cell count slope was the most strongly correlated. This indicates that the rate of increase or decrease of CD4 cell count at a given time-point was the parameter that was most strongly associated with current VL. However, the distributions of ΔCD4 cell count and CD4 cell count slope values were very broad even among patients who maintained virological suppression. This suggests that considerable fluctuations in CD4 cell counts occur among patients despite sustained virological control. When these distributions were compared with the distributions of data from patients who had current virological failure, they almost completely overlapped. This demonstrated that absolute CD4 cell counts and CD4 cell count changes could not be used to identify patients who have developed virological failure. These findings were further corroborated by the observation that the distributions of CD4 cell count changes in the 30 patients who never achieved virological suppression were also broadly overlapping with the distributions of data from those who maintained virological suppression.

To investigate these associations further, we focussed on the use of CD4 cell count slopes since this was the parameter most strongly associated with VL at a group level. However, ROC curve analysis confirmed that use of CD4 slopes provided very poor test characteristics for predicting virological failure. The specificity and sensitivity of a negative CD4 cell count slope was low, showing that this parameter was not of practical utility in this clinical setting. Furthermore, the data show that a negative CD4 cell count slope could not even be used as a screen to identify those at high risk of virological failure as a means of rationing scarce viral load monitoring resources.

A strength of this study is that patients were closely followed in a multicentre clinical trials unit with strict protocols for regular clinical and laboratory monitoring every 2–3 months, leading to reliable identification of virological failure. As soon as a VL > 1,000 copies/ml was first detected, confirmatory viral load testing was done. The cohort characteristics were diverse and so the data are not only relevant to those with advanced immunodeficiency. Despite differing cohort characteristics, follow-up procedures and analytic approaches, our data are consistent with and extend previous studies that have found a poor association between CD4 cell counts and the development of virological failure [8,15].

We acknowledge the limitations of this study. An important potential limitation is that all patients studied were ART-naïve. Therefore these findings may not be generalisable to treatment-experienced patients. Our patients participated in international multicentre clinical trials. Their experience may differ from that of patients accessing treatment in a community-based setting. We do not have good assessments of treatment compliance although the mechanism underlying virological failure is unlikely to affect the relationship between CD4 cell counts and viral load. Despite a limited cohort size, follow-up in this study was prolonged, a substantial proportion developed virological failure and the number of paired CD4 cell counts and VL measurements was large.

## Conclusion

In conclusion, we have shown that although changes in CD4 cell count correlated significantly with VL at a group level, they had very poor predictive value when being used to assess individual patients. Thus, CD4 cell count measurements cannot be used as a substitute for virological failure monitoring. Rigorous cost benefit analyses are required to further evaluate use of VL monitoring in this setting. Furthermore, there is a great need for development of simplified techniques to measure VL and for exploration of alternative low-cost assays for monitoring [26].

## Competing interests

The authors declare that they have no competing interests.

## Authors' contributions

All the authors participated in the design of the analyses and the writing and revising of the manuscript. MB did the analyses.

## Pre-publication history

The pre-publication history for this paper can be accessed here:


